# Gas volume corrections in intensive care unit: needed or pointless?

**DOI:** 10.1152/japplphysiol.00225.2023

**Published:** 2023-06-22

**Authors:** Rosanna D'Albo, Federica Romitti, Luigi Camporota, Onnen Moerer, Mattia Busana, Luciano Gattinoni

**Affiliations:** ^1^Department of Anesthesiology, University Medical Center Göttingen, Göttingen, Germany; ^2^Department of Adult Critical Care, Guy’s and St Thomas’ NHS Foundation Trust, Health Centre for Human and Applied Physiological Sciences, London, United Kingdom

**Keywords:** dead space, extracorporeal support, gas volumes, intensive care, venous admixture

## Abstract

The conditions of temperature, pressure, and saturation in which respiratory gas volumes are expressed [standard temperature and pressure, dry (STPD), ambient temperature and pressure, saturated (ATPS), or body temperature and pressure, saturated (BTPS)] are physiologically relevant, but often ignored or unknown in clinical practice. In this study, we aimed to investigate whether and at which extent the gas volume corrections, either in natural or artificial lung, may alter key respiratory and metabolic variables and the possible clinical consequences. We primarily referred to the effects of gas volume corrections on three physiological variables: physiological dead space, venous admixture, and total CO_2_ production (V̇co_2_) during extracorporeal support. We used three physiological models in which calculations of these variables have been performed with and without correction of gas volumes, both in a theoretical model and in 448 patients. The lack of gas volume correction leads to an error in the computation of physiological dead space fraction between 0.05 and 0.15, both in the theoretical model and in the patient population. The venous admixture was minimally affected by the absence of correction (0.01–0.04 error). During extracorporeal support, if the V̇co_2_ of natural and membrane lung is expressed in different conditions, potentially large errors (0%–18.4%) may occur in the computation of total V̇co_2_ (V̇co_2tot_ = V̇co_2ML_ + V̇co_2NL_). This may lead to inappropriate settings of mechanical ventilation with higher plateau pressure. As the dead space and the CO_2_ sharing between natural and artificial lung are relevant both as prognostic index and as a guide for appropriate mechanical ventilation, their inappropriate computation may lead to erroneous categorization of the patients and inappropriate mechanical treatment.

**NEW & NOTEWORTHY** Gas volume conditions are often ignored or unknown in the clinical practice. However, they could have relevance for the calculation of some key variables in ICU setting. This study shows that gas volume corrections are mostly relevant when assessing CO_2_ clearance, both in mechanical ventilation and during extracorporeal support, whereas irrelevant for oxygenation assessment of patients. Knowing when the appropriate corrections are needed allows to better understand patients’ clinical conditions and to tailor the treatment.

## Graphical abstract

**Figure:**
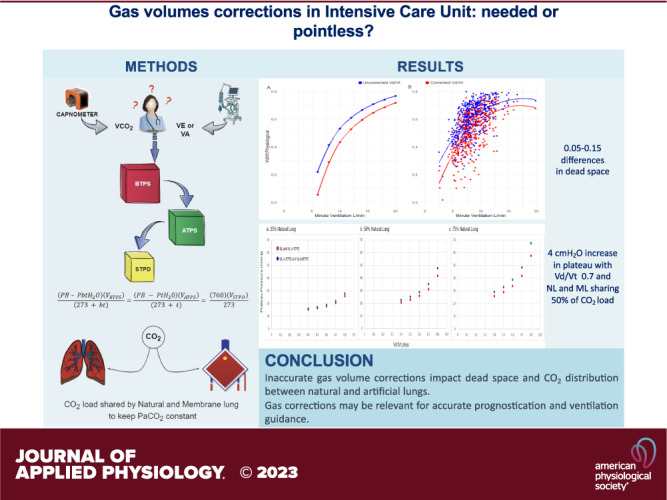

## INTRODUCTION

The expression of gas volumes in standard conditions, either body temperature and pressure, saturated (BTPS) or standard temperature pressure, dry (STPD), is a usual procedure in physiology, as it was in the critical care literature of the 1970s (1). More recently, gas volumes are reported in the literature without any indication of the conditions in which they are measured or expressed, and some variables are measured at BTPS, such as the gas volumes derived from the ventilator, and others in STPD, such as the gas volumes derived from indirect calorimetry or the volumetric capnography. Therefore, some respiratory variables [e.g., dead space, ventilatory efficiency, minute ventilation (V̇e), CO_2_ production (V̇co_2_)] combine different parameters measured at different conditions (e.g., gases measured in the blood or gases measured by the ventilator or spirometer), but no indication is offered regarding the conditions of measurement or whether a conversion has been performed to ensure accurate and reproducible results.

This issue not only has theoretical importance but can have implication when threshold values are used to make diagnoses, stratify disease severity, or determine eligibility to clinical trials if physiological enrichment is considered (2).

Regardless of the specific conditions in which some of the gas volumes are expressed, it is important to understand the consequences that may result from comparing gas volumes measured at different conditions [i.e., BTPS for ventilation and STPD for CO_2_ elimination or oxygen consumption (V̇o_2_)] and the errors that may result from the lack of correction.

In this study, we aim to quantify the effect of gas volume correction on the computation of key respiratory variables, such as physiological dead space and venous admixture, during mechanical ventilation. In addition, we will focus on the possible clinical consequences (3, 4) of lack or inappropriate use of gas volume corrections during extracorporeal membrane oxygenation (ECMO) (5) or extracorporeal CO_2_ removal (ECCO_2_R; 6, 7).

## MATERIALS AND METHODS

A narrative theoretical explanation of the problem, with practical examples of transformations according to the laws of gases, is presented in the online Supplemental Material: https://doi.org/10.6084/m9.figshare.22578514. Here, we limit our presentation to the basic equations used to generate the results.

### Definitions

ATPS **=** ambient temperature (273 Kelvin + ambient temperature in Celsius), atmospheric pressure (mmHg), saturated (i.e., in the presence of water vapor at that temperature).

STPD = standard temperature (273 Kelvin), atmospheric pressure (760 mmHg), dry (i.e., absence of water vapor).

BTPS **=** body temperature (273 Kelvin + body temperature in Celsius), atmospheric pressure, saturated (i.e., in the presence of water vapor at a given temperature).

Considering the three conditions (i.e., ATPS, STPD, and BTPS), the following equations apply, all of them based on Boyle–Mariotte’s and Charles’ laws (1, 8):

$$\frac{\left(PB\;-\;PbtH_{2}O\right)\left(V_{BTPS}\right)}{\left(273\;+\;bt\right)}=\frac{\left(PB\;-\;PtH_{2}O\right)\left(V_{ATPS}\right)}{\left(273\;+\;t\right)}=\frac{\left(760\right)\left(V_{STPD}\right)}{273},$$

where PB is the barometric pressure, PbtH_2_O is the water pressure at body temperature, PtH_2_O is the water vapor pressure at the temperature at which the gas is measured, bt is the body temperature, and *t* is the temperature at which the gas is actually measured. It follows that:

$$V_{BTPS}=V_{ATPS}\frac{\left(PB\;-\;PtH_{2}O\right)}{\left(273\;+\;t\right)}\times \frac{\left(273\;+\;bt\right)}{\left(PB\;-\;PbtH_{2}O\right)}.$$

Therefore, for example, to convert V̇co2 from ATPS to BTPS, the barometric pressure must be known, as well as the temperature and the humidity, from which the vapor pressure may be derived. In practice, the barometric pressure is usually assumed to be 760 mmHg and the PbtH_2_O is assumed to be 47 mmHg (i.e., the water vapor pressure at 37°C of body temperature). The V̇co_2_ corrected at BTPS is then divided for the minute ventilation (V̇e) or alveolar ventilation (VA) to obtain the correct fraction of gas (both in BTPS). Indeed, V̇e and VA are usually measured at BTPS. Once the correct fraction is obtained, being the gases expressed in the same condition, the correspondent CO_2_ partial pressures [mixed expired CO2 pressure (${\text{P}\bar{\text{E}}}_{{\text{CO}}_{\text{2}}}$) for minute ventilation and alveolar CO_2_ pressure ($\text{P}{\text{A}}_{{\text{CO}}_{2}}$) for alveolar ventilation, respectively] are computed by multiplying the gas fraction times the dry pressure present in the alveoli (i.e., PB − PbtH_2_O). This is 713 mmHg, assuming a barometric pressure of 760 mmHg and body temperature of 37°C:

$$P{\bar{\text{E}}}_{\text{C}{\text{O}}_{2}}=\frac{\dot{V}\text{c}{\text{o}}_{2}\text{× }(\text{Pb}-\text{Pbt}{\text{H}}_{2}O)}{\dot{\text{V}}E}\;,\text{P}{\text{A}}_{\text{C}{\text{O}}_{2}}=\frac{\dot{V}\text{c}{\text{o}}_{2}\text{× }(\text{Pb}-\text{Pbt}{\text{H}}_{2}O)}{\text{V}A},$$

where V̇co_2_ and V̇e and/or VA are expressed in the same conditions. Note that the exact measurement implies the measurement of barometric pressure, to which the PEEP (if any) expressed in millimeter(s) of mercury should theoretically be added.

### Physiological Dead Space

#### Model.

We simulated the variations of physiological dead space (Vd/Vt_phys_) in relation to variations of minute ventilation (V̇e) from 5 to 20 L/min. The Vd/Vt was calculated using the Bohr’s equation (9):

$$\frac{\text{Vd}}{\text{Vt}}=\frac{\text{P}{\text{A}}_{\text{C}{\text{O}}_{2}}-\text{P}{\bar{\text{E}}}_{\text{C}{\text{O}}_{2}}}{\text{P}{\text{A}}_{\text{C}{\text{O}}_{2}}}\text{.}$$

The $\text{P}{\text{A}}_{\text{C}{\text{O}}_{2}}$  was kept constant at 40 mmHg, whereas the ${\text{P}\bar{\text{E}}}_{{\text{CO}}_{\text{2}}}$ was computed in two different ways, to simulate an erroneous calculation and then the corrected one, while keeping the V̇co_2_ constant at 0.25 L/min at STPD:

$$\text{Uncorrected }P{\bar{\text{E}}}_{\text{C}{\text{O}}_{2}}=\frac{\dot{\text{V}}\text{C}{\text{O}}_{\text{2STPD}}\times 713}{\dot{\text{V}}E_{\text{BTPS}}}\;\text{,}$$

in which V̇e is expressed as usually measured by the ventilator (BTPS) and V̇co_2_ is expressed in STPD as usually in capnometry.

$$\text{Corrected }P{\bar{\text{E}}}_{\text{C}{\text{O}}_{2}}=\frac{\dot{V}\text{C}{\text{O}}_{\text{2BTPS }}\text{× 713}}{\dot{\text{V}}E_{\text{BTPS}}}\;\text{,}$$

in which V̇e and V̇co_2_ are expressed in the same conditions (BTPS).

#### Study population.

We applied the same approach to 448 consecutive adults (>18 yr) with acute hypoxemic respiratory failure due to COVID-19 pneumonia, admitted to the Department of Adult Critical Care at Guy’s and St Thomas’ NHS Foundation Trust, London, UK, between March 2020 and March 2021. This retrospective study was registered in the United Kingdom as a service evaluation (number 10796) with a waiver of consent for the use of anonymized retrospective data accrued though provision of routine clinical services. All procedures were performed in accordance with the ethical standards of the responsible committee on human experimentation and with the Helsinki Declaration of 1975. Data were anonymized locally before entry into the central database.

To calculate Vd/Vt_phys_, in the absence of directly measured $\text{P}{\text{A}}_{\text{C}{\text{O}}_{2}}$ and ${\text{P}\bar{\text{E}}}_{{\text{CO}}_{2}}$, we used the formula as described by Beitler et al. (10) to assess the errors deriving from using the uncorrected and corrected Vd/Vt_phys_:

$$\text{Uncorrected}\frac{\text{Vd}}{\text{V}{\text{t}}_{\text{phys}}}=\frac{\text{VD}}{\text{VT}}=\text{1}-\frac{\text{713}\times \dot{\text{V}}\text{C}{\text{O}}_{\text{2STPD}}}{\left(\dot{V}E_{\text{BTPS}}\text{ × P}{\text{a}}_{\text{C}{\text{O}}_{2}}\right)}.$$

$$\text{Corrected}\frac{\text{Vd}}{\text{V}{\text{t}}_{\text{phys}}}=\frac{\text{VD}}{\text{VT}}=\text{1}-\frac{\text{713 × }\dot{\text{V}}\text{C}{\text{O}}_{\text{2BTPS}}}{\left(\dot{V}E_{\text{BTPS }}\text{× P}{\text{a}}_{\text{C}{\text{O}}_{2}}\right)}$$

Further details are available in the Supplemental Material.

### Venous Admixture Model

Two variables (gas volume dependent) may affect the computation of venous admixture: respiratory quotient (RQ) and capillary venous O_2_ content (CvO_2_).

Keeping the V̇o_2_ in STPD before applying the conversion at 0.3 L/min and the V̇co_2_ at 0.25 L/min, the RQ has been computed as follows:

$$\text{Uncorrected RQ }=\frac{\dot{V}\text{C}{\text{O}}_{\text{2STPD}}}{\dot{V}O_{\text{2BTPS}}},$$

in which V̇co_2_ and V̇o_2_ are expressed in two different conditions.

$$\text{Corrected RQ }=\frac{\dot{V}\text{C}{\text{O}}_{\text{2STPD}}}{\dot{V}O_{\text{2STPD}}},$$

in which V̇co_2_ and V̇o_2_ are expressed in the same condition.

The two derived RQs have been then used for the computation of two different alveolar partial pressure of oxygen ($\text{P}{\text{A}}_{{\text{O}}_{2}}$) (the uncorrected and the corrected one), which, in turn, entered in the computation of an uncorrected and a corrected capillary content of oxygen (CcO_2_), as follows:

$$P{\text{A}}_{O_{2}}=\text{ F}{\text{I}}_{O_{2}}\text{× 713}-\text{P}{\text{A}}_{\text{C}{\text{O}}_{2}}\times \frac{\dot{V}O_{2}}{\dot{V}\text{C}{\text{O}}_{2}}.$$

$$\text{Cc}{\text{O}}_{2}\;=\left(\text{Sa}{\text{t}}_{\text{c}}\text{× 1.39 × Hb}\right)\text{ + }(\text{0.003 × P}A_{O_{2}}).$$

V̇o_2_ either in STPD or in BTPS has been used for the calculation of two different CvO_2_ (the uncorrected and the corrected one) as follows:

$$\text{Cv}O_{2}=\text{ C}{\text{a}}_{O_{2}}-\frac{\dot{V}O_{2}}{Q},$$

where Q is the cardiac output and CaO_2_ is the arterial O_2_ content, computed as follows:

$$CaO_{2}=\left(Sat_{a}\times 1.39\times Hb\right)+(0.003\times \text{P}{\text{a}}_{\text{O}}{}_{{}_{2}}).$$

We set the following inputs: $FI_{O_{2}}$ = 21%, $\text{P}{\text{A}}_{\text{C}{\text{O}}_{2}}$ = 45 mmHg, Sat_c_ = 100%, Hb = 15 g/dL, and Q = 5 L/min. We had the arterial O_2_ pressure ($\text{P}{\text{a}}_{\text{O}}{}_{{}_{2}}$) varied from 20 to 120 mmHg and we derived the Sat_a_ from Kelman’s equation (11).

Venous admixture has been then calculated as follows:

$$Venous\;admixture\;fraction=\frac{CcO_{2}\;-\;CaO_{2}}{CcO_{2}\;-\;CvO_{2}}.$$

### Extracorporeal Support Model

We used a model to investigate the variations of V̇co_2_ when applying the different gas volume corrections. We simulated the V̇co_2_ variations from natural lung, artificial lung, as well as the resulting total V̇co_2_ (V̇co_2Total_ = V̇co_2_ natural lung + V̇co_2_ membrane lung). The absolute and relative differences between the total V̇co_2_ obtained from different conditions have been then computed. The corresponding error in minute ventilation (ΔVE) to maintain a constant ${\text{P}\bar{\text{E}}}_{{\text{CO}}_{\text{2}}}$ (40 mmHg in our model) has been calculated as follows:

$$\Delta \dot{\text{V}}\text{E }=\;\frac{\dot{\Delta \text{V}}\text{C}{\text{O}}_{2}}{\text{P}{\bar{\text{E}}}_{\text{C}{\text{O}}_{2}}}.$$

The consequent plateau pressure values potentially used to adjust for the gas correction were calculated as follows:

$$P_{\text{plat}}=\text{PEEP}-\frac{\dot{V}\text{C}{\text{O}}_{2}}{\text{Crs }\times \;\text{RR }\times \;\frac{P{\bar{\text{E}}}_{\text{C}{\text{O}}_{2}}}{\text{713}}\;\times \;(\frac{\text{Vd}}{\text{Vt}}-1)}.$$

See Supplemental Material for further details.

## RESULTS

### CO_2_ Clearance

**Figure 1.**
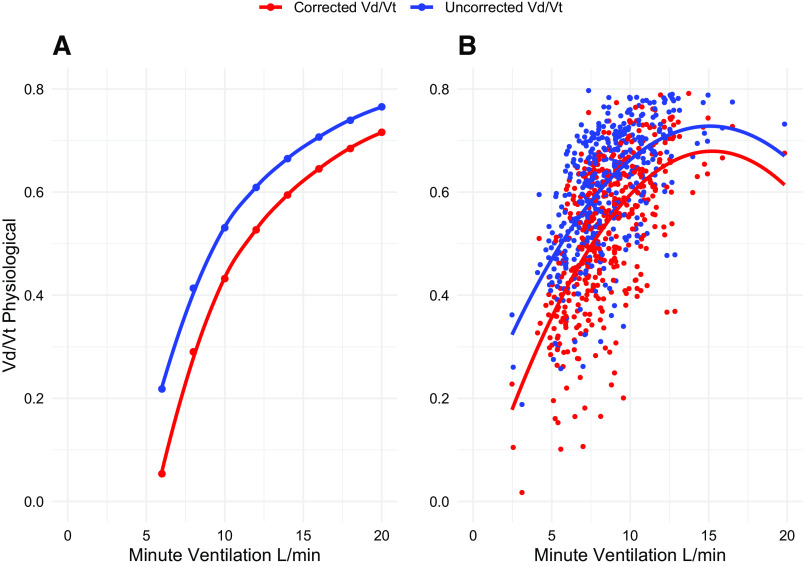
Vd/Vt_phys_ is shown as a function of V̇e. *A*: Vd/Vt from physiological model. *B*: data from the real patient population. Vd/Vt_phys_, physiological dead space; V̇e, minute ventilation.

**Figure 2.**
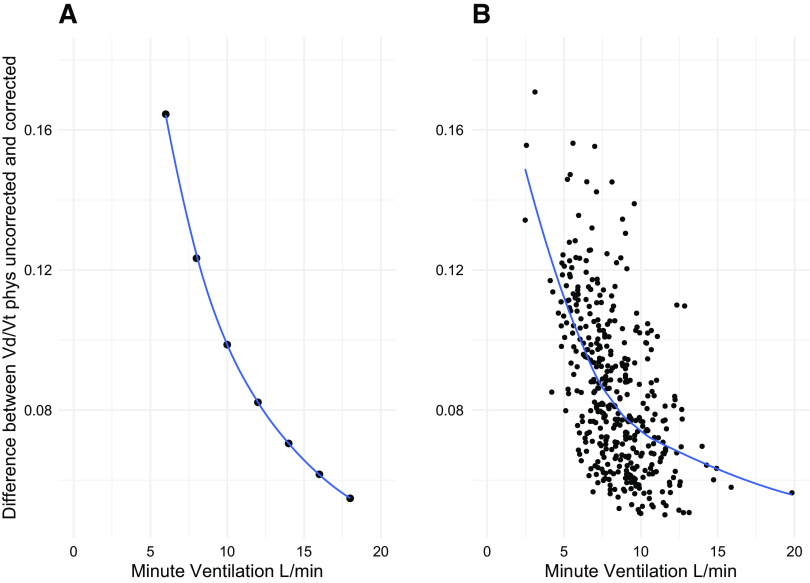
Absolute difference between uncorrected and corrected Vd/Vt_phys_ is shown as a function of total ventilation both in the model (*A*) and in the real patient population (*B*). Vd/Vt_phys_, physiological dead space.

In Fig. 1, we represent Vd/Vt_phys_ as a function of total ventilation. In Fig. 1*A*, we show the Vd/Vt_phys_ after the proper gas correction (i.e., both V̇co_2_ and V̇e expressed at the same condition of pressure and temperature)—red circles and the Vd/Vt_phys_ when the correction is not performed—blue circles (i.e., V̇e expressed at BTPS and V̇co_2_ at STPD). In Fig. 1*B*, we show the Vd/Vt_phys_ computed from real patients’ data with and without gas correction. As shown, a remarkable and reproducible difference in Vd/Vt_phys_ may be observed both in the model and in the patient population. This absolute difference decreases with the increasing V̇e, from 0.15 to 0.05 (at lower and higher V̇e, respectively), both in the model and in the real patient population (Fig. 2). The main anthropometric and clinical patients’ characteristics are reported in Table 1.

**Table 1.** Main anthropometric and clinical characteristics of the 448 patient population

Variable	Median and IQR
Age	59 (51–66)
Sex M, %	71
Height, m	1.75 (1.65–1.76)
Actual weight, kg	81 (75–95.25)
BMI, kg/m^2^	27.7 (24–32.87)
Plateau pressure, cmH_2_O	24 (21–27)
Peak pressure, cmH_2_O	26 (23–29)
Respiratory rate, beats/min	18 (16–22)
Tidal volume, mL	460 (400–516)
V̇co_2,_ mL/min	190.6 (173.6–213.19
Physiological dead space fraction	0.55 (0.43–0.64)
$FI_{O_{2}}$, %	60 (50–80)
$\text{P}{\text{a}}_{\text{O}}{}_{{}_{2}}$/$FI_{O_{2}}$	109 (87–146)
$Pa_{CO}{}_{{}_{2}}$	44.3 (38.5–50.4)
Mechanical power, J/min	14.6 (11.1–19.8)
Entry SOFA score	6 (4–7)
Mortality, %	32

BMI, body mass index; $FI_{O_{2}}$, fraction of inspired oxygen; IQR, interquartile range; $Pa_{CO}{}_{{}_{2}}$, arterial CO_2_ pressure; $\text{P}{\text{a}}_{\text{O}}{}_{{}_{2}}$, arterial O_2_ pressure; SOFA, sequential organ failure assessment; V̇co_2_, CO_2_ production.

### O_2_ Consumption and Oxygenation Variables

**Figure 3.**
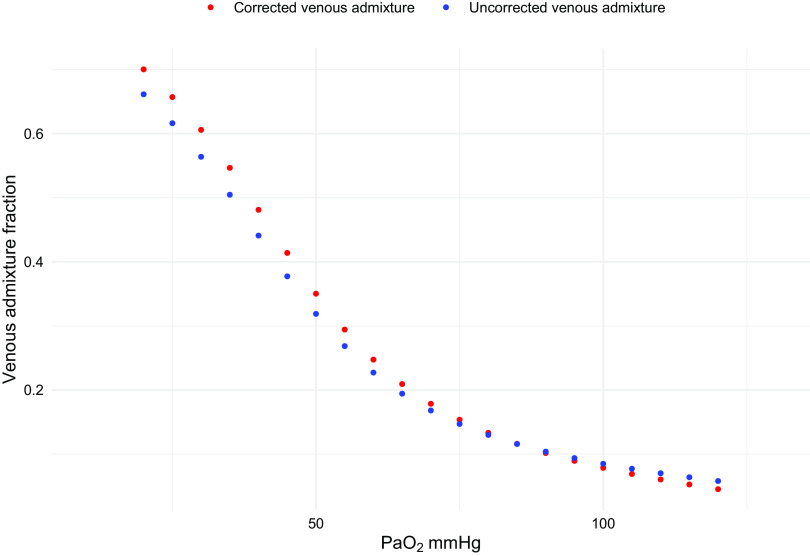
Venous admixture is shown as a function of $\text{P}{\text{a}}_{\text{O}}{}_{{}_{2}}$. Red circles represent venous admixture computation with correction, whereas blue circles show venous admixture computation without correction. $\text{P}{\text{a}}_{\text{O}}{}_{{}_{2}},$ arterial O_2_ pressure.

Most of the oxygenation variables may be affected by the presence or absence of gas volume corrections. If the V̇o_2_ in BTPS is matched with a V̇co_2_ obtained by the ventilator in STPD, the resulting respiratory quotient (RQ) is artificially altered (see Supplemental Fig. S2), and this will introduce errors in the calculation of energy requirements (see Supplemental Fig. S3) and in the alveolar gas equation, leading to significant errors in the determination of alveolar PO_2_ (see Supplemental Fig. S4,). This in turn will alter the computation of variables like $\text{P}{\text{a}}_{\text{O}}{}_{{}_{2}}$/$\text{P}{\text{A}}_{{\text{O}}_{2}}$ ratio and the computation of venous admixture, a key variable for oxygenation assessment. In Fig. 3, we show the venous admixture computed with gas correction (blue circles) and the venous admixture computed without gas correction (red circles) as a function of $\text{P}{\text{a}}_{\text{O}}{}_{{}_{2}}$. The absolute difference ranges from 0.04 to 0.01.

### Effects on Extracorporeal Support

The extracorporeal support is a technique allowing the sharing of CO_2_ load between natural and artificial lungs, being V̇co_2Total_ = V̇co_2_ natural lung + V̇co_2_ membrane lung. An incorrect partitioning of CO_2_ will lead to inadequate ventilator setting or false estimation of the contribution of extracorporeal support to the total CO_2_ elimination.

In Table 2, we report the V̇co_2_ values from the membrane and the natural lungs, when expressed at different conditions and their possible combinations. As shown, the error deriving from the erroneous partitioning of V̇co_2_ between artificial and membrane lungs can be various. This may lead to possible errors in the computation of minute ventilation and, in turn, in the ventilator setting. For the sake of clarity, we report here two examples, which we consider the closest to reality.

1.  V̇co_2_ from artificial lung in STPD and V̇co_2_ from natural lung in BTPS, compared with both V̇co_2_ in STPD.
2.  V̇co_2_ from artificial lung in ATPS and V̇co_2_ from natural lung in STPD, compared with both V̇co_2_ in STPD.

**Table 2.** Absolute and relative differences in computing V̇CO_2TOT_ during extracorporeal support

Conditions		% ML	% NL	V̇co_2ML_	V̇co_2NL_	V̇co_2TOT_, L/min	Delta L/min	Diff %	Delta vs. STPD-STPD, L/min	Diff % vs. STPD-STPD
ML	NL									
**STPD**	**STPD**	**75**	**25**	**0.187**	**0.062**	**0.25**	**0**	**0**	**0**	**0**
		**50**	**50**	**0.125**	**0.125**	**0.25**	**0**	**0**	**0**	**0**
		**25**	**75**	**0.062**	**0.187**	**0.25**	**0**	**0**	**0**	**0**
STPD	BTPS	75	25	0.187	0.076	0.263	0.013	5.20	0.013	5.2
		50	50	0.125	0.151	0.276	0.026	10.40	0.026	10.4
		25	75	0.062	0.227	0.289	0.039	15.60	0.039	15.6
STPD	ATPS	75	25	0.187	0.069	0.256	0.006	2.40	0.006	2.4
		50	50	0.125	0.137	0.262	0.012	4.80	0.012	4.8
		25	75	0.062	0.206	0.268	0.018	7.20	0.018	7.2
**ATPS**	**ATPS**	**75**	**25**	**0.206**	**0.069**	**0.275**	**0**	**0**	**0.025**	**10**
		**50**	**50**	**0.137**	**0.137**	**0.274**	**0**	**0**	**0.024**	**9.6**
		**25**	**75**	**0.069**	**0.206**	**0.275**	**0**	**0**	**0.025**	**10**
ATPS	STPD	75	25	0.206	0.062	0.256	−0.019	−6.91	0.006	2.4
		50	50	0.137	0.125	0.262	−0.012	−4.38	0.012	4.8
		25	75	0.069	0.187	0.268	−0.007	−2.55	0.018	7.2
ATPS	BTPS	75	25	0.206	0.076	0.282	0.007	2.55	0.032	12.8
		50	50	0.137	0.151	0.288	0.014	5.11	0.038	15.2
		25	75	0.069	0.227	0.296	0.021	7.64	0.046	18.4
**BTPS**	**BTPS**	**75**	**25**	**0.207**	**0.076**	**0.283**	**0**	**0**	**0.033**	**13.2**
		**50**	**50**	**0.138**	**0.151**	**0.289**	**0**	**0**	**0.039**	**15.6**
		**25**	**75**	**0.069**	**0.227**	**0.296**	**0**	**0**	**0.046**	**18.4**
BTPS	STPD	75	25	0.207	0.062	0.269	−0.014	−6.92	0.019	7.6
		50	50	0.138	0.125	0.263	−0.026	−11.15	0.013	5.2
		25	75	0.069	0.187	0.256	−0.04	−4.83	0.006	2.4
BTPS	ATPS	75	25	0.207	0.069	0.276	−0.007	−2.47	0.026	10.4
		50	50	0.138	0.137	0.275	−0.014	−4.84	0.025	10
		25	75	0.069	0.206	0.275	−0.021	−7.09	0.025	10

The table shows the conditions in which membrane lung (ML) and natural lung (NL) can be expressed, their percentage of CO_2_ load, and their correspondent V̇co_2_. The V̇co_2TOT_ is equal to V̇co_2ML_ + V̇co_2NL_. The differences (absolute − “Delta L/min” and relative − “Diff %”) are computed in two ways: comparing V̇co_2TOT_ time by time to when V̇co_2_ from ML and V̇co_2_ from NL is measured in the same condition (i.e., STPD-STPD, BTPS-BTPS, ATPS-ATPS; bold rows); comparing V̇co_2TOT_ always to when V̇co_2_ from both the natural and the membrane lungs is measured in STPD. ATPS, ambient temperature and pressure, saturated; BTPS, body temperature and pressure, saturated; STPD, standard temperature and pressure, dry; V̇co_2_, CO_2_ production.

**Figure 4.**
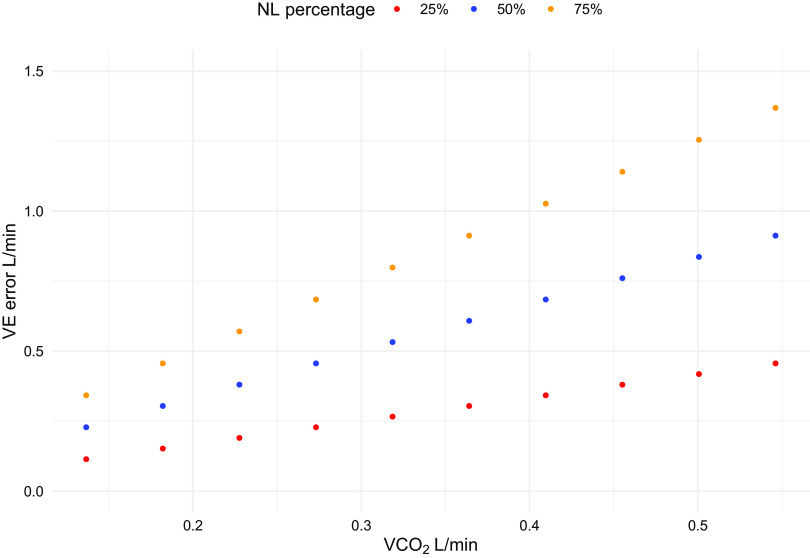
The error in minute ventilation when considering natural lung (NL) in BTPS and membrane lung (ML) in STPD during extracorporeal support is shown as a function of V̇co_2_. Three different circumstances (75%, 50%, 25% of the total CO_2_ load taken by natural lung, respectively) are represented. BTPS, body temperature and pressure, saturated; STPD, standard temperature and pressure, dry; V̇co_2_, CO_2_ production.

**Figure 5.**
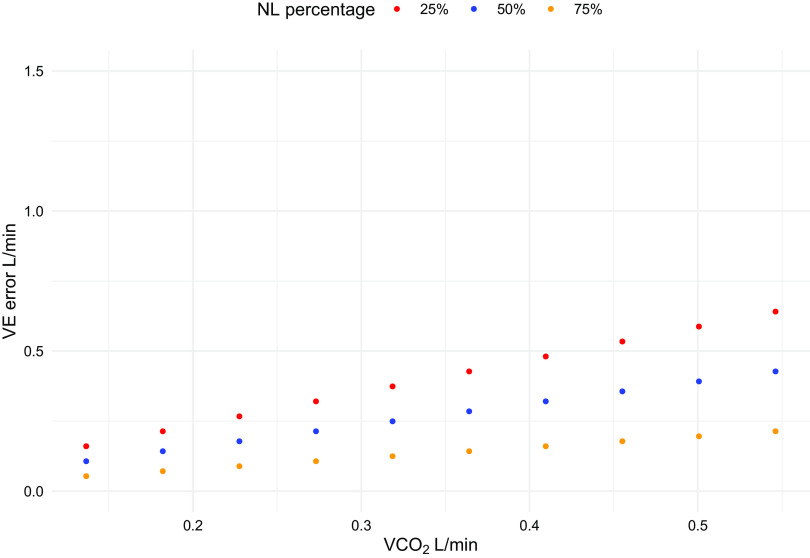
The error in minute ventilation when considering natural lung (NL) in STPD and membrane lung (ML) in ATPS during extracorporeal support is shown as a function of V̇co_2_. Three different circumstances (75%, 50%, 25% of the total CO_2_ load taken by natural lung, respectively) are represented. ATPS, ambient temperature and pressure, saturated; STPD, standard temperature and pressure, dry; V̇co_2_, CO_2_ production; V̇e, minute ventilation.

In both cases, the total V̇co_2_ will be larger, although to a different extent, than the one computed when taking into account both V̇co_2_ in STPD. The error in minute ventilation [keeping the arterial CO_2_ pressure ($\text{P}{\text{a}}_{\text{C}\text{O}}{}_{{}_{2}}$) constant] will change as illustrated in Figs. 4 and 5. As shown, in both circumstances, it increases with increasing total V̇co_2_, but it has opposite behavior: in the first case, the error is greater the more the CO_2_ load is taken by the natural lung and in the second case, the error is greater the less the CO_2_ load depends on the natural lung. This is explained by the fact that in the first example, the V̇co_2_ from natural lung (and consequently the V̇e) is overestimated compared with the one from the artificial lung, so that the more the natural lung “works” the greater the error. In the second example, it is the membrane lung V̇co_2_ to be overestimated, so that the error will decrease with the natural lung working more.

**Figure 6.**
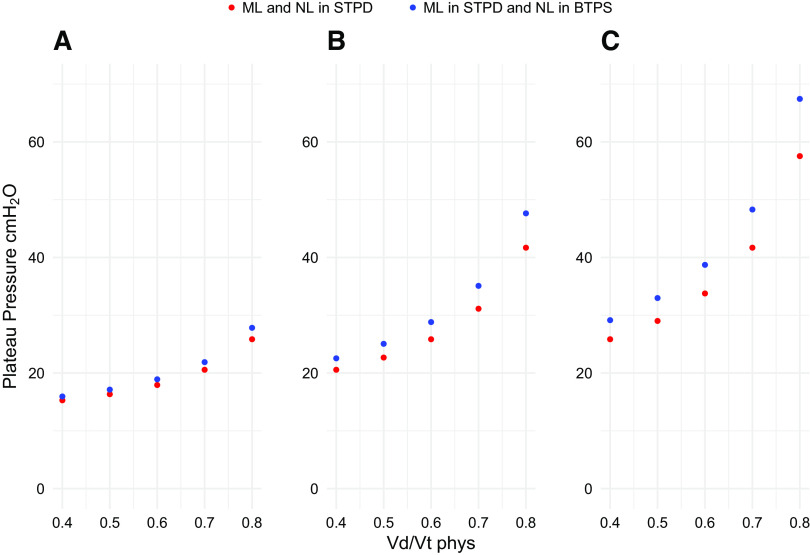
The effects on plateau pressure, taking into account the V̇co_2_ from natural and artificial lungs in different conditions (BTPS and STPD, respectively), are shown as a function of Vd/Vt_phys_. *A*–*C*: three circumstances in which the natural lung shares the 25%, 50%, and 75% of the total V̇co_2_, respectively. ML, membrane lung; NL, natural lung; Vd/Vt_phys_, physiological dead space.

The increased minute ventilation due to the erroneously overestimated natural lung V̇co_2_ in the first example would unavoidably lead to an increase of tidal volume and/or respiratory rate. This will increase the mechanical power (12) and, if the respiratory rate is kept constant, to an increase of driving and plateau pressures. The consequences are shown in Fig. 6, where we simulated different CO_2_ sharing between artificial and natural lungs during the weaning from extracorporeal support.

## DISCUSSION

The main finding of this study is that the lack of the appropriate correction of gas volumes may lead to

1.  Significant errors in dead space estimation.
2.  Smaller error in the estimation oxygenation variables (e.g., venous admixture).
3.  Errors in assessing dependency and ventilatory settings in patients on extracorporeal support.

### Physiological Dead Space

An overestimation of dead space due to lack of correction may lead to two main consequences. First, as the dead space is a variable with great prognostic value in patients with ARDS (13), its overestimation may lead to falsely assign a patient to a different prognostic category and potentially include or exclude from interventions or clinical trials. Second, and more important, the dead space, more than oxygenation, is the variable that most reflects the structural changes of the lung (14). Indeed, with time, in the unsolved respiratory failure, although the oxygenation levels remain usually stable, the dead space progressively increases, associated with increased fibrosis, septal disruptions, and microthrombosis (15, 16). Finding, as an example, erroneously 65% dead space instead of 50% may lead physicians to a different therapeutical approach, based on misjudgment of the underlying lung structural condition. Elevated $\text{P}{\text{a}}_{\text{CO}}{}_{{}_{2}}$ with dead space of 50% instead of 65% may reflect, instead of structural changes, metabolic changes as an increased V̇co_2_ production and oxygen consumption, which could require a different management of the patient (3). In summary, a correct analysis of the ventilatory relationships within V̇co_2_, dead space, and minute ventilation is needed for a correct assessment of the underlying respiratory and metabolic status, which may be in part altered by a misleading computation of these key determinants (17).

### Oxygenation

Variables that may be affected by the lack of adequate correction of gas volumes are the respiratory quotient and the computed central venous oxygen content. As the V̇o_2_ is one of the determinants of $\text{P}{\text{A}}_{{\text{O}}_{2}}$, which, in turn, determines the capillary oxygen content and the venous admixture, any increase of V̇o_2_ from STPD to BTPS, being the V̇co_2_ constant, would decrease the respiratory quotient. Such a decrease, for a given $\text{P}{\text{A}}_{{\text{CO}}_{2}}$, leads to an increase of computed $\text{P}{\text{A}}_{{\text{O}}_{2}}$ and an increase of the computed capillary oxygen content. At the same time, however, if the central venous oxygen content is computed from a V̇o_2_ considered either in BTPS or STPD, a lack of correction would lead to a misjudgment in this calculation. As in the venous admixture computation, the capillary and the central venous oxygen content would play in opposite directions, the effects of miscalculation would partially offset each other, resulting in minor changes in the final oxygenation assessment (see Fig. 3).

### Extracorporeal Support

A particular attention to gas volume corrections must be paid during extracorporeal support, a technique whose use is increasing worldwide, including during the recent pandemic (18). Indeed, the key point of these techniques, both considering ECMO and ECCO_2_R, is not as much the effect on oxygenation, but the possibility of tailoring more gentle mechanical ventilation by an appropriate sharing of CO_2_ clearance load between artificial and natural lungs. Although, surprisingly, rarely reported, the knowledge of the CO_2_ cleared naturally or artificially is a basic principle for tailoring appropriate mechanical ventilation. This is particularly true during weaning from extracorporeal support, to correctly assess whether the natural lung may safely support the total CO_2_ clearance, being the contribution of artificial lung progressively reduced down to zero. An example of a possible error derives from a recent study we performed during ECMO weaning (4). Indeed, if erroneously a part of the CO_2_ clearance is attributed to the natural lung instead of the artificial one, the ventilator variables to maintain an adequate $\text{P}{\text{a}}_{\text{CO}}{}_{{}_{2}}$ would be set at an erroneous level. In patients in which respiratory failure is so severe to require extracorporeal support, even a modest change in ventilatory setting requirements may lead to a potentially harmful mechanical ventilation. In our model, as an example, a 0.03 L/min increase in V̇co_2_ for the natural lung leads to ∼4 cmH_2_O increase in plateau pressure, for a VD/VT_phys_ equals to 0.7, when natural and membrane lung both share half of the total V̇co_2_ (see Fig. 6*B*). The plateau pressure may further increase up to 7–10 cmH_2_O during the weaning phase, when the CO_2_ cleared from the membrane lung is 25% (see Fig. 6*C*). This has to be taken into account when deciding whether the patient can be actually weaned from extracorporeal support. Given that the criteria for weaning from extracorporeal support are still not well defined and largely vary in the literature reports (19–22), a precise definition of the CO_2_ load to be actually eliminated from the natural lung may help in the correct assessment of weaning criteria.

### Conclusions

In summary, our theoretical paper includes results that were partly validated using data obtained from a real population. We show that the absence of gas volume correction could potentially lead to significant clinical consequences, particularly in severe clinical conditions. This issue primarily pertains to patient stratification and mechanical ventilation settings. In general, most of the research in intensive care is increasingly reliant on epidemiological/statistical methodologies and there is a progressing trend aimed at simplifying the physiological measurements and approach to the patients. However, strong and accurate physiological foundations are necessary for the treatment of individual patients. The lack of knowledge in the pathophysiological mechanisms will hinder, by definition, a personalized approach to diagnosis and treatment. In turn it requires precise assessment and accurate measurements, which include the standardized calculation and correction of gas volumes.

## DATA AVAILABILITY

Data will be made available upon reasonable request.

## SUPPLEMENTAL DATA

10.6084/m9.figshare.22578514 Supplemental Material and Supplemental Figures: https://doi.org/10.6084/m9.figshare.22578514.

## DISCLOSURES

No conflicts of interest, financial or otherwise, are declared by the authors.

## AUTHOR CONTRIBUTIONS

R.D. and L.G. conceived and designed research; R.D. and L.G. analyzed data; R.D., F.R., L.C., and L.G. interpreted results of experiments; R.D. prepared figures; R.D. and L.G. drafted manuscript; F.R., L.C., M.B., and L.G. edited and revised manuscript; R.D., F.R., L.C., O.M., M.B., and L.G. approved final version of manuscript.
